# What is gold standard and what is ground truth?

**DOI:** 10.1590/2176-9451.19.5.027-030.ebo

**Published:** 2014

**Authors:** Jefferson Rosa Cardoso, Ligia Maxwell Pereira, Maura Daly Iversen, Adilson Luiz Ramos

**Affiliations:** 1 Associate Professor, State University of Londrina (UEL); 2 Department of Physical Education, State University of Londrina (UEL); 3 Professor and Chair, Department of Physical Therapy, Movement and Rehabilitation Sciences, Northeastern University; Section of Clinical Sciences, Division of Rheumatology, Immunology & Allergy, Brigham & Women's Hospital, Harvard Medical School, Boston and Department of Women and Children's Health, Karolinska Institute, Stockholm, Sweden; 4 Associate Professor, Department of Dentistry, State University of Maringá (UEM)

*"What has not been examined impartially, has not been well examined. Scepticism is therefore the first step towards truth."* (Denis Diderot, Philosopher) 

Clinical decision-making is complex and based upon accurate evaluation of clinical findings using diagnostic tests and reference standard data. Given that many aspects of dental examination are not direct measures, but rely on indirect measures, it is important for clinicians to understand the basic principles and terms used to assess the accuracy of diagnostic tests and to appropriately evaluate published literature regarding these tests. Luckily, there is a variety of readily available metric systems to assess the quality of diagnostic test studies and to help clinicians better understand evidence-based literature. 

Dentistry, or shall we say Clinical Dentistry, is becoming more complex and patients have been better informed. Importantly, health care has also shifted focus to emphasize evidence-based practice (EBP). EBP is considered the gold standard for health professional decision-making. No one can deny that the activities in the field of evidence-based Dentistry have grown exponentially in the last decade. However, we cannot forget that Pierre Fauchard (1678 - 1761) may have been the first to warn the dental field about the concept of evidence, taking into consideration the practices of the time. Fauchard and James Lind (1716-1790) were both concerned about the health of sailors dying of scurvy and, for this reason, conceptualized a "clinical trial" involving the use of vitamin C to counteract the disease. The former even tested techniques for the removal of caries, dental restoration and implants.

The true meaning of evidence-based Dentistry is grounded in a solid understanding and application of clinical epidemiology principles to reduce any confusion that may exist due to academic training. Epidemiology is defined as the "Science of making predictions about individual patients or a group, by recounting clinical events in similar patients in order to ensure that the predictions are correct". Clinical epidemiology is "a subfield that applies the principles and methods of epidemiology to study the occurrence and outcomes of disease in people with a given illness".^1^

The ability to precisely define a question of interest (clinical question), derive relevant information from databases, differentiate research methodology, select statistical procedures as well as the ability to critically evaluate studies and understand their implications for care, are required skills.^2^ However, let us not be too optimistic; there are drawbacks. Ironically, political, social and economic pressure limits the time available for practitioners to seek answers to clinical questions. Furthermore, there is a surprising number of weekly published studies, from the best to the worst.

This paper will discuss a clinical question, among several that can be built "epidemiologically", specifically, diagnostic test accuracy. In other words, the study will provide estimates of the ability of a diagnostic test to discriminate between patients with or without a pre-defined health condition, comparing the results with a standard reference test. There will always be one predictor variable (result of the test) and an outcome (presence or absence of the disease).^3^ Furthermore, we add the concept of ground truth, which is a set of measures known to be more accurate than the measurements of the system you are testing.

The term gold standard refers to a benchmark that is the available under reasonable conditions. Indeed, is not the perfect test, but merely the best available one that has a standard with known results. This is especially important when faced with the impossibility of direct measurements.^4^ In Dentistry, for example, micro computed tomography can be considered a gold standard for the diagnosis of proximal carious lesions of posterior teeth, as microscopic examination of the enamel has demonstrated its acuracy.^5^ In the past, referring to an examination as the gold standard meant that it was unqualifiedly the most accurate procedure. However, in present clinical practice, even though the intent of term has not changed, its use is dependent upon the context of the statistical method being used.

A gold standard study may refer to an experimental model that has been thoroughly tested and has a reputation in the field as a reliable method. The correct interpretation of a diagnostic test demands one to master specific concepts such as sensitivity, specificity, prevalence, positive and negative predictive values. The sensitivity of a test is defined as the proportion of people with the inherent disease who test positive (true-positive). The specificity of a test is the proportion of people without the disease that have a negative test (true-negative). In some literature, one can find the term 1-specificity that is defined as the rate of false positives (in other words, the percentage of the sample incorrectly identified as positive). Typically, a Receiver Operating Characteristic curve (ROC) is used as a graphical representation of the rate of sensitivity and specificity. The area under the curve represents the accuracy of the test. The closer the value is to one, the greater the test accuracy. In many clinical scenarios, there is a trade off between sensitivity and specificity. This trade off is related to the fact that some people will clearly be normal while others will have the condition. However, there will inevitably be a group of patients who fall in a middle zone (neither clearly normal nor abnormal). In such instances, an arbitrary cut off will be used to distinguish between normal and abnormal. Any screening test used to distinguish between patients in this circumstance will have a trade off between sensitivity and specificity. One way to address this dilemma is to use a combination of diagnostic tests to develop a diagnosis.

Positive predictive value is the probability of patients with true positive results (they have the condition of interest) to test positive. Negative predictive value, on the other hand, is defined as the probability of patients with true negative results (no disease) to test negative. It is important to recognize that diagnostic tests are influenced by the prevalence of the disease in the population being tested. Prevalence is the probability of an individual to have the disease (based on clinical characteristics and demographic data) in a population and includes both newly diagnosed cases and existing cases. Likelihood ratio is the ratio between the probability of a particular outcome of a diagnostic test in individuals with the disease and the probability of that same outcome in individuals without the disease. This may be positive or negative.^6^

To best understand how and why diagnostic tests function, a basic understanding of Bayes theorem is needed. Bayes defined probability as "the ratio between the value at which an expectation depending on the happening of the event ought to be computed, and the value of the thing expected upon its happening".^7^ For example, the probability a person has to be diagnosed with oral cancer and having a positive test for the condition depends not only on the relationship between events, but also on the accuracy of the test and the prevalence of the condition in the population sample. Thus, if one wishes to evaluate the operating characteristics of a diagnostic test and selects a sample consisting of only a few people with oral cancer, whereas another individual evaluates the same diagnostic test in a sample with a greater proportion of people with oral cancer, test sensitivity, specificity, positive and negative predictive values may vary considerably even though the test procedure was identical.^8^

An ideal diagnostic method hypothetically presents a sensitivity of 100% with respect to detection of injury or illness (identifying all cases of injury or disease in all specimens evaluated or individuals with no false negatives) and a specificity of 100% (without false positives, pointing to injury or illness where there is none). Thus, in practice, there is no perfect gold standard. Instead, we have a method with the greatest sensitivity and the highest specificity. Therefore, the gold standard diagnostic of the past has probably been changed today.

Higher sensitivity values increase negative predictive values. Higher specificity values increase positive predictive values. Thus, if the test has higher values of sensitivity and specificity, all people having a positive test result have the disease, while all patients who have a negative test do not have the disease. Therefore, there is a trade off between these values. This concept is important in instances in which the diseases have a poor prognosis. In these cases, one might want the test to have higher sensitivity so as not to unduly distress patients with lots of false positive results. Alternatively, if a disease is easily treatable, it might be more important to screen the population at risk by means of a test with less sensitivity and higher specificity. For patients who are a false positive, a second test can be used to confirm diagnosis.^9^

For example, in Medicine, angiography (arteriography) by contrast was a former gold standard for heart disease. A recent study reported the sensitivity of angiography to be 66.5% and the specificity to be 82.6%. Now magnetic resonance angiography (MRA) has become the new gold standard, with a reported sensitivity of 86.5% and a specificity of 83.4%.^10^ The acceptance of a new gold standard default method takes time and exhaustive evidence, especially if the internal validity is consistent and acceptable. 

As for ground truth, it can signify the mean value from the collection of data from a particular experimental model (that preferentially uses gold standard method) representing behavioral reference. For example, using an universal shear testing machine to evaluate the strength of a new resin for bracket bonding, we obtain a value of X. This value can be compared to a reference value obtained by previous observations. Thus, if the resulting X value is similar to or higher than those found in ground truth, it can be said that this new resin has an appropriate value. There is a consensus that the clinical resistance pattern for bracket bonding corresponds to something around 6.8 Mpa (this value matches more in ground truth definition than gold standard as it can not be precisely checked).^11^ So this value can be used as reference ground truth to accept or reject the hypothesis that a particular new resin has admissible clinical strength or resistance. Therefore, in simple terms, a gold standard test refers to a diagnostic method with the best accuracy; whereas ground truth represents the reference values used as standard for comparison purposes.

In a recent study, authors classified midpalatal suture ossification in five maturation stages.^12^ A total of 140 cone-beam computed tomography (CBCT) scans from palatal suture were collected and blindly classified into five stages. The images were used as ground truth reference. Subsequently, 30 images were randomly evaluated and reclassified by three experienced orthodontists. The authors found strong agreement in the proposed classification method, with kappa index ranging from 0.82 to 0.93. However, for this diagnostic method of suture maturation to become a gold standard, histological confirmation is required to test specificity and sensibility. In other words, it should be tested whether CBCT scans of "no suture" really mean midpalatal suture tissue absence or the opposite in their five stages.

When a clinician or researcher is interested in critiquing a study, which describes the process for evaluating a diagnostic test, or conducting such study, it is important to note that studies of a diagnostic test follow the rules described in the literature. The *Standards for Reporting of Diagnostic Accuracy Studies* (STARD)^13^ is a list containing 25 items used to critically evaluate the quality of a particular diagnostic test study. Another accepted format used to evaluate studies of diagnostic tests is the *Quality Assessment of Studies of Diagnostic Accuracy Included in Systematic Reviews* (QUADAS).^14^ the latter is a 14-item checklist (answers can be "yes", " no" or" unclear ") used to measure potential risk of bias in systematic reviews. Systematic reviews of these studies may follow the format proposed by the Cochrane Collaboration available at (Cochrane* Handbook for Systematic Reviews of Diagnostic Test Accuracy*) (http://srdta.cochrane.org/handbook-dta-reviews).

In conclusion, gold standard data or method is related to something that has already been checked (histologically, microscopically, chemically, etc.) and presents the best accuracy (sensitivity and specificity). Ground truth means data and/or method related to more consensus or reliable values/aspects that can be used as references, but were not or cannot be checked. We recommend more exposure to concepts of clinical epidemiology in dental schools to ensure the best evidence-based practice.
